# Research progress of single-cell sequencing in tuberculosis

**DOI:** 10.3389/fimmu.2023.1276194

**Published:** 2023-10-13

**Authors:** Jiahui Pan, Zecheng Chang, Xinyue Zhang, Qinzhou Dong, He Zhao, Jingwei Shi, Guoqing Wang

**Affiliations:** Key Laboratory of Pathobiology Ministry of Education, College of Basic Medical Sciences/China-Japan Union Hospital of Jilin University, Jilin University, Changchun, China

**Keywords:** single-cell sequencing, scRNA-seq, tuberculosis, *Mycobacterium tuberculosis*, host-pathogen interaction

## Abstract

Tuberculosis is a major infectious disease caused by *Mycobacterium tuberculosis* infection. The pathogenesis and immune mechanism of tuberculosis are not clear, and it is urgent to find new drugs, diagnosis, and treatment targets. A useful tool in the quest to reveal the enigmas related to *Mycobacterium tuberculosis* infection and disease is the single-cell sequencing technique. By clarifying cell heterogeneity, identifying pathogenic cell groups, and finding key gene targets, the map at the single cell level enables people to better understand the cell diversity of complex organisms and the immune state of hosts during infection. Here, we briefly reviewed the development of single-cell sequencing, and emphasized the different applications and limitations of various technologies. Single-cell sequencing has been widely used in the study of the pathogenesis and immune response of tuberculosis. We review these works summarizing the most influential findings. Combined with the multi-molecular level and multi-dimensional analysis, we aim to deeply understand the blank and potential future development of the research on *Mycobacterium tuberculosis* infection using single-cell sequencing technology.

## 1 Introduction

*Mycobacterium tuberculosis* (*Mtb*) is an obligate aerobic microorganism (1). Tuberculosis (TB) is an infectious disease caused by *Mtb* infection and remains a major global cause of mortality (2). In 2021, there were 10.6 million new TB cases worldwide, and China is among the countries burdened with high TB incidence (3). TB primarily spreads through respiratory aerosols and can affect various organs systemically, with pulmonary tuberculosis being the most common manifestation (4). Currently, the clinical diagnosis of pulmonary tuberculosis largely relies on chest radiography, sputum smear microscopy, and bacteriological cultures (5). Rapid diagnostic techniques such as auramine-rhodamine fluorescence and the AccuProbe *Mycobacterium tuberculosis* complex test have also been widely utilized (6, 7). However, the ever-evolving public health challenges necessitate novel diagnostic tools and therapeutic approaches. Additionally, there is a growing need to gain deeper insights into the impact of *Mtb* infection on the host and the underlying immune mechanisms.

Although traditional molecular biology experimental methods have successfully revealed certain aspects of the infection and immune mechanisms of *Mtb*, such as surfactant protein D located in the alveoli which can inhibit *Mtb* infection by blocking mannose oligosaccharide residues on the bacterial cell surface (8), *Mtb* can secrete the virulence factor Rv0222 to escape the immune response after infecting the human body (9). However, after infection, a comprehensive panorama of the intricate host immune regulatory processes and dynamic evolution still lacks elucidation. Our understanding of the diversity and heterogeneity of immune cells, as well as their relevant physiological, pathological processes, and molecular mechanisms, remains incomplete. Therefore, there is an urgent need to explore the mechanisms of *Mtb* infection and the dynamic host immune processes at the single-cell level, to identify novel drug targets and new immunotherapeutic approaches.

In this section, we provide a concise overview of the development of single-cell sequencing. We place particular emphasis on the discoveries gathered through the application of single-cell sequencing in the field of tuberculosis research, as reported in the literature. Additionally, we discuss the research domains that, in our view, merit exploration through the utilization of single-cell sequencing methodologies.

## 2 Single-cell sequencing

Traditional transcriptomic analyses have been employed to comprehend the pathogenesis, but single-cell sequencing offers novel perspectives and significant convenience in enhancing our understanding of human cell heterogeneity, identifying susceptible cell types, and elucidating infection dynamics (10, 11). Single-cell sequencing is a high-throughput sequencing analysis performed at the individual cell level, encompassing transcriptomic, genomic, and epigenomic profiling. It bridges certain limitations of traditional sequencing techniques and molecular experiments, unraveling the genetic architecture and gene expression states of individual cells while reflecting intercellular heterogeneity (12). Consequently, single-cell sequencing technology has emerged as a highly precise, reliable, and efficient analytical approach in recent years, playing a crucial role in research domains such as oncology and virology (13, 14).

### 2.1 Single-cell RNA sequencing

**Figure 1:**
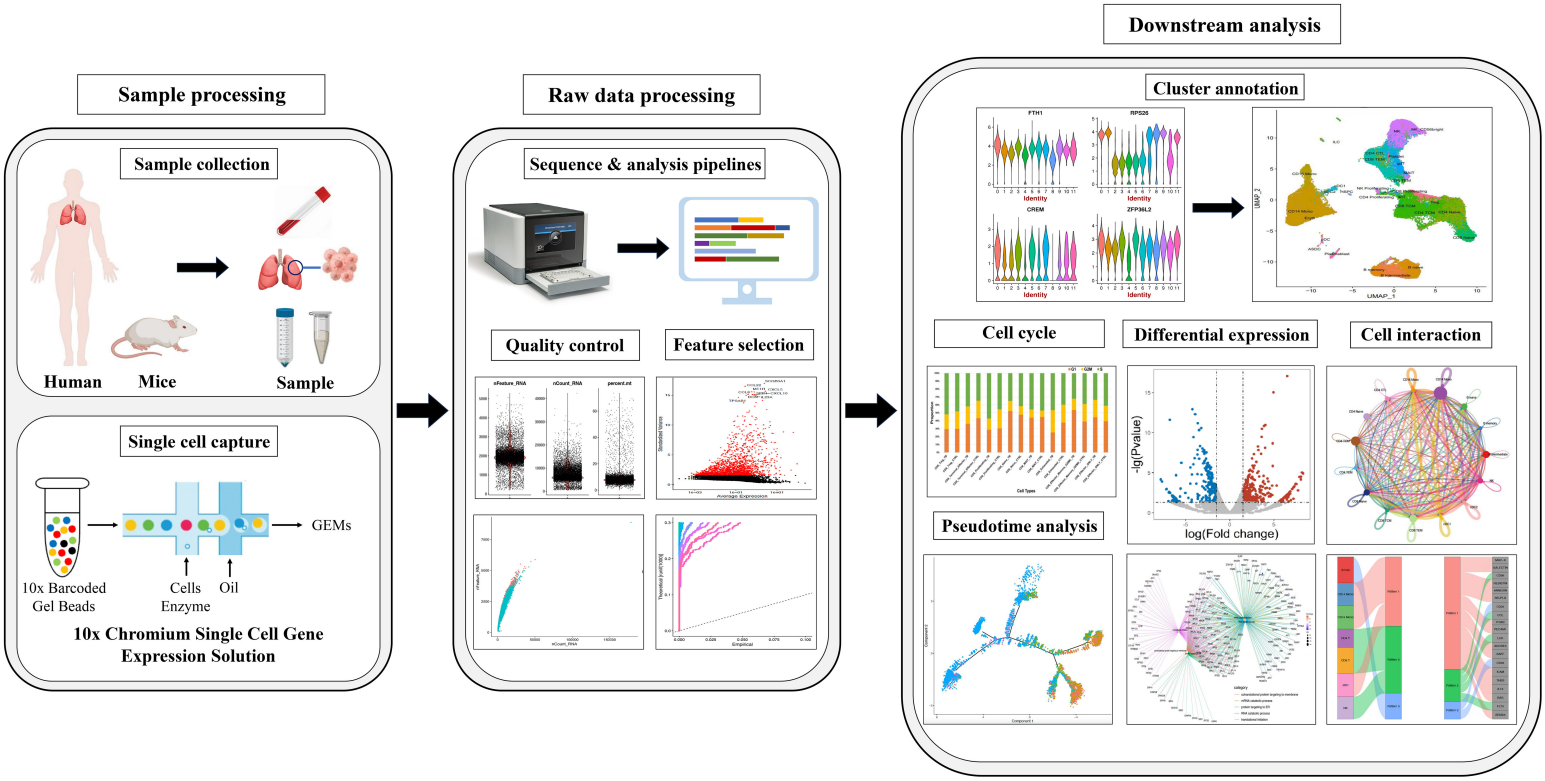
The main procedure for single-cell RNA sequencing.

Since the inception of single-cell transcriptomic sequencing at the individual cell level which was proposed by Professor Fuchou Tang (15), researchers have embarked on a new era of single-cell sequencing. Among these techniques, single-cell transcriptomic sequencing (scRNA-seq) stands out as the most widely applied approach. The technique has evolved from conventional transcriptomics methodologies. In conventional transcriptomics studies, the typical workflow entails RNA extraction, reverse transcription to generate cDNA, sequencing and library construction. The principle of this technology is commencing with high-throughput cellular capture and labeling, followed by transcriptome amplification to procure transcriptional information. Subsequently, comprehensive data analysis is conducted to extract meaningful insights (16).This technique enables single-cell level analysis of millions of cells within a single study. Despite the minute amount of starting material in each cell, it allows for cell classification, qualitative characterization, and discrimination at the single-cell resolution, thereby identifying rare cell populations (17). Generally, scRNA-seq steps include sample processing, single-cell capture, sequence and analysis pipelines, quality control, feature selection, cluster annotation, cell cycle, pseudotime analysis, differential expression and cell interaction (**Figure 1**).

**Figure 2:**
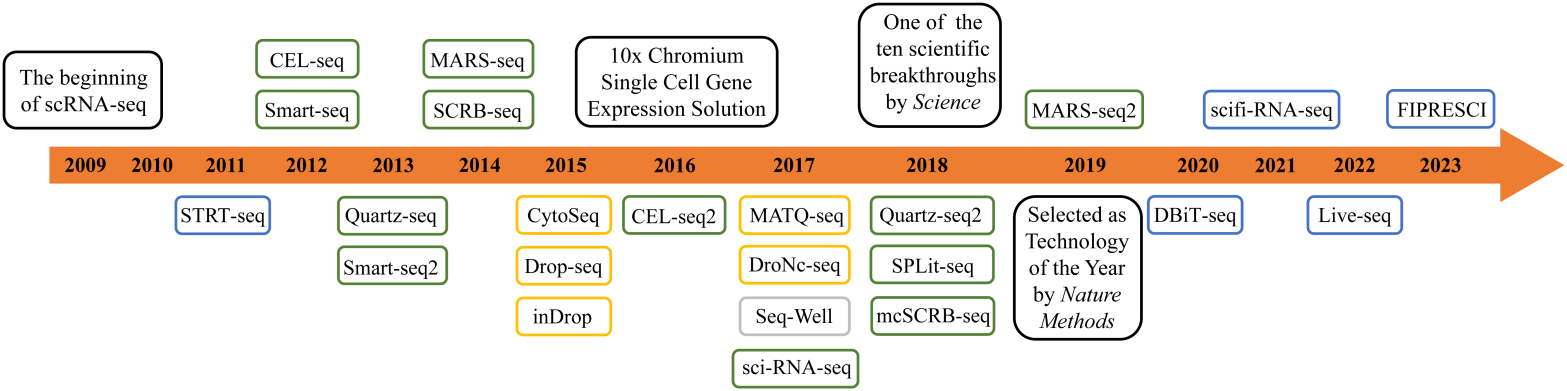
The development of scRNA-seq technology. The blue boxes represent microfluidics-based technology. The green boxes represent plate-based technology. The orange boxes represent microdroplet-based technology. The grey box represents nanowell-arrays-based technology.

In 2011, Islam et al. developed single-cell tagged reverse transcription (STRT-Seq) upon previous research. This technology enabled the identification of transcript 5′-end samples and analysis of promoter usage within single cells, thereby increasing the number of detectable cells and the diversity of cell types that could be analyzed (18). In 2012, Daniel Ramsköld et al. developed the Switching mechanism at the 5' end of the RNA transcript (Smart-Seq), which enhanced the read coverage of the transcriptome, thereby facilitating detailed analysis of alternative transcript isoforms (19). In the same year, Tamar Hashimshony et al. addressed the limitation of low starting RNA amounts in scRNA-seq by developing Cell Expression by Linear amplification and Sequencing (CEL-seq), a method that can prove beneficial for transcriptomic analysis of complex tissues containing multiple cell types (20). In 2013, building upon prior research, Simone Picelli et al. introduced Smart-seq2, a technique that significantly enhanced the sensitivity and accuracy of single-cell transcriptomic analysis, allowing for comprehensive profiling of the transcriptome at the individual cell level (21). In 2014, Islam et al. introduced an alternative quantification standard in the realm of scRNA-seq known as the unique molecular identifier(UMI). UMI is a single-strand region with a randomly synthesized nucleotide with a length ranging from 4 to 12. It is incorporated into primers before reverse transcription, enabling the unique barcoding of the 5' or 3' end of each mRNA copy of a transcript. Leveraging UMI information allows for enhanced quantification of mRNA molecules, effectively mitigating the errors introduced by PCR, and giving rise to UMI-based absolute quantification transcriptomic sequencing (22). In the subsequent years, there has been an exponential surge in related techniques for scRNA-seq, including Quartz-seq (23), MARS-seq (24), Drop-Seq (25), MARS-seq2.0 (26), inDrop (27), and others (**Figure 2**, **Supplementary Table 1**).

### 2.2 ATAC-seq

In eukaryotes, chromatin serves as the fundamental genetic unit, typically composed of DNA, histones, non-histone proteins, and a small amount of RNA. Chromatin represents a dynamic nuclear structure that undergoes transformation into chromosomes during both mitosis and meiosis. During the interphase of the cell cycle, it exhibits transcriptional activity (28). Chromatin plays a pivotal role in regulating the expression of cell-specific genes (29, 30), and the investigation of chromatin-associated nuclear protein structures also aids in elucidating how epigenetic factors control gene expression (31). From the conventional Chromatin Immunoprecipitation (ChIP) to the chromatin immunocleavage (ChIC), research methodologies in the field of epigenetics have been continuously evolving (32, 33). As scRNA-seq has unlocked the sequencing potential at single-cell resolution, researchers have concurrently delved into the development and exploration of single-cell epigenomic sequencing techniques. Among the more commonly employed methods are MNase-seq, DNase-seq, and NOMe-seq (34, 35).

However, due to the aforementioned issues such as complex protocols, poor reproducibility, the requirement for a large number of cells, and suboptimal sequencing signals, Jason D Buenrostro and colleagues introduced the Assay for Transposase-Accessible Chromatin with high throughput sequencing (ATAC-seq) in 2013. This method utilizes engineered Tn5 transposase coupled with high-throughput sequencing technology to map nucleosome positioning and investigate chromatin accessibility at the single-cell resolution (36). ATAC-seq steps are composed of cell nuclei preparation, transposition, and amplification (37). This technique requires a relatively low sample quantity, significantly reducing the required experimental time. Moreover, due to the non-cutting nature of Tn5 transposase, it can capture complete information on the accessible regions. However, the presence of mitochondrial DNA may still introduce some errors during the detection process.

### 2.3 Single-cell genome sequencing

Single-cell genome sequencing is a sophisticated technique which can amplify minute quantities of the entire genome DNA at the single-cell level, followed by exome capture and sequencing. This technology enables researchers to obtain a high coverage of the complete genome, allowing for comprehensive analysis of the genetic information of each cell. The main purpose of this technique is to investigate the impact of individual cells on biological processes, uncover differences among cell populations, and reveal the evolutionary relationships between cells (38). The primary workflow of this technique involves several key steps: isolation of single cells, whole-genome amplification, detection of the amplified genome, error correction to minimize potential inaccuracies, and determination of the genetic relationships among individual cells (38). In response to the need for the isolation of individual cells, various methods have been developed, including microfluidic and bead-based methods (39) and microwell dilution (40). Currently, using droplets or micromechanical valves in microfluidic device has become more prevalent (41, 42). In the step of whole-genome amplification, various methods such as multiple displacement amplification (MDA) (43), degenerate oligonucleotide primed PCR (DOP-PCR) (44), and eMDA (45) have been utilized. However, the most commonly employed method is multiple annealing and looping-based amplification cycles (MALBAC), which was introduced in 2012 (46). This technique utilizes random primers and novel temperature cycling and it requires a smaller amount of template. Compared to other technologies, MALBAC requires a smaller amount of template, and its amplification uniformity significantly surpasses that of other amplification methods. Over 90% of loci can be successfully amplified, enabling accurate detection of copy number variations (47).

### 2.4 Spatial transcriptomics

The majority of techniques involve enzymatic dissociation of tissues to obtain single cells, leading to the loss of spatial positional information during the research process. To overcome this limitation, researchers have developed Spatial transcriptomics, which is based on scRNA-seq. Spatial transcriptomics aims to analyze the transcriptional profile information from different spatial locations within the sample (48). Currently, there are two main approaches for spatial transcriptomics detection: 1) imaging-based methods; 2) sequencing-based methods (49).

Imaging-based methods primarily refer to techniques that are based on imaging mRNAs *in situ* via microscopy. Based on the method of mRNA discrimination, two approaches can be distinguished (50). The first one is *in situ* hybridization (ISH), which involves the hybridization of mRNAs to fluorescently labeled, gene-specific probes. Single-molecule RNA fluorescence *in situ* hybridization (smFISH) is a highly classic ISH method, which allows for quantitative assessment of the target samples (51). However, this method has limitations in terms of the number of fluorescent labels it can accommodate, resulting in suboptimal performance when multiple fluorescent labels are required (52). With the advancement of technology, various techniques such as seqFISH (53), seqFISH+ (54), MERFISH (55), and enhanced electric FISH (56) have been developed and expanded upon the foundation of smFISH. These methods have increased the detectable quantity of targets and improved the stability of fluorescent signals. Another *in situ* approach is the *in situ* sequencing-based(ISS-based) technologies. This method enables direct sequencing of mRNA from fixed tissue or cellular samples, allowing the discovery of associations between sequencing information and its spatial location (e.g., subcellular localization) (52). The first ISS probe was introduced by Rongqin Ke and colleagues in 2013 and is known as padlock probes, which are single-strand molecules of DNA complementary to the target cDNA (57). In the subsequent advancements, various methods such as barcode *in situ* targeted sequencing (BaristaSeq) (58), fluorescence *in situ* sequencing (FISSEQ) (59), and STARmap (60) have been applied in spatial transcriptomics-related research.

The sequencing-based methods primarily involve the direct extraction of mRNA from tissues while preserving the associated spatial information for sequencing. The earliest related detection using this approach was the “spatial transcriptomics” developed by Patrik L Ståhl (48). By positioning histological sections on arrayed reverse transcription primers with unique positional barcodes, Patrik L Ståhl and colleagues successfully obtained high-quality RNA sequencing data with spatial positional information from mouse brain tissue and human breast cancer tissue. Similar techniques based on this principle include: Slide-seq, a method for transferring RNA from tissue sections onto a surface covered in arrays composed of 10 μm-diameter DNA-barcoded beads with known positions, allowing the locations of the RNA to be inferred by sequencing (61); High-definition spatial transcriptomics (HDST), a spatial transcriptomics technique with 2μm spatial resolution. This method captures RNA from histological tissue sections on a dense, spatially barcoded bead array, enabling higher resolution spatial transcriptome sequencing (62).

Combining the previous achievements of single-cell sequencing in fields such as HIV (63), SARS-CoV-2 (64), and Salmonella (65), it is evident that single-cell sequencing plays a crucial role in deciphering the pathogenic mechanisms and exploring therapeutic targets for related diseases. By investigating and refining specific cell populations associated with pathogen infections at the cellular and molecular levels, followed by the identification of genes and phenotypes regulated by these cells, and further uncovering the associations with immune-related genes, we can infer the phenotypes of these genes, elucidate their relevance to the host immune response, and map an immune regulatory atlas. These methods provide new insights into the *Mtb*-host interaction, as well as the diagnosis and treatment strategies for related diseases.

## 3 Findings from single-cell sequencing applications in TB research

**Figure 3:**
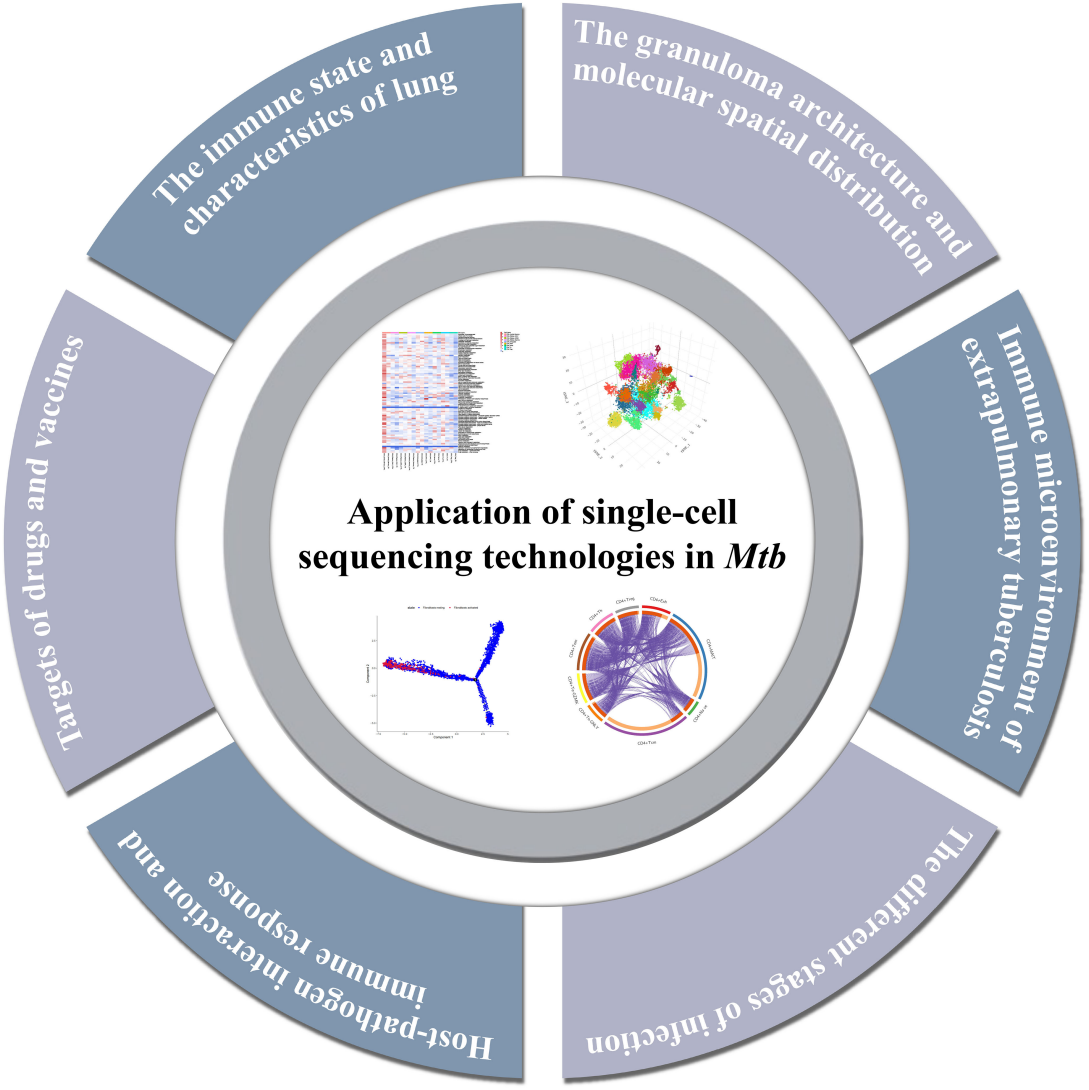
Summary of the major findings resulting from single-cell sequencing application in tuberculosis research.

Previous analysis methods may have neglected some rare cell groups, but these few cells are probably the key factors to determine the tissue state (66). The use of single-cell sequencing can not only avoid this situation, but also identify susceptible cell types, changes in immune status, find biomarkers, and detect intercellular variation of hosts. It helps us better understand the interaction between the host and *Mtb*, and finally develop drugs and vaccines and develop new treatment strategies. Throughout this section, the main findings using single-cell sequencing in the field of tuberculosis will be explored (**Figure 3**, **Table 1**).

**Table 1:** Overview of current single-cell sequencing studies in tuberculosis.

Author	Title	Journal	Host	Key findings	Ref
Gierahn, Todd M et al., 2017	Seq-Well: portable, low-cost RNA sequencing of single cells at high throughput	*Nature methods*	Mouse and Human	Genetic characteristics of thousands of primitive human macrophages exposed to tuberculosis were analyzed by Seq-Well.	(67)
Bossel Ben-Moshe, Noa et al., 2019	Predicting bacterial infection outcomes using single cell RNA-sequencing analysis of human immune cells	*Nature communications*	Human	It develop a dynamic deconvolution algorithm, which provides a predictive power to identify risk factors for human infectious disease	(68)
Cai, Yi et al., 2020	Single-cell transcriptomics of blood reveals a natural killer cell subset depletion in tuberculosis	*EBioMedicine*	Human	The frequency change of CD3-CD7+GZMB+ subgroup can distinguish patients with TB from LTBI and HC	(69)
Khan, Nargis et al., 2020	*M. tuberculosis* Reprograms Hematopoietic Stem Cells to Limit Myelopoiesis and Impair Trained Immunity	*Cell*	Mouse	It indicates that *Mtb* accesses the BM to target innate immunity by imprinting HSCs with a unique transcriptomic profile that suppresses myelopoiesis and impairs innate immune control of *Mtb* infection.	(70)
Pisu, Davide et al., 2021	Single cell analysis of M. tuberculosis phenotype and macrophage lineages in the infected lung	*The Journal of experimental medicine*	Mouse and Human	It shows show that the main macrophage populations in the lung are epigenetically constrained in their response to infection, while inter-species comparison reveals that most AMs subsets are conserved between mice and humans.	(71)
Nathan, Aparna et al., 2021	Multimodally profiling memory T cells from a tuberculosis cohort identifies cell state associations with demographics, environment and disease	*Nature immunology*	Human	It profile memory T cells from a TB progression cohort at post-disease immune steady state and multimodally define cell states associated with demographics, environment, and TB progression.	(72)
Cronan, Mark R et al., 2021	A non-canonical type 2 immune response coordinates tuberculous granuloma formation and epithelialization	*Cell*	Zebrafish and Rhesus	It find that, rather than an exclusively type 1 inflammatory response, there is induction of both type 1 and type 2 responses within the granuloma.	(73)
Esaulova, Ekaterina et al., 2021	The immune landscape in tuberculosis reveals populations linked to disease and latency	*Cell host & microbe*	Rhesus	Characterization of lung landscape during tuberculosis and control using single cells and conventional techniques.	(74)
Gideon, Hannah P et al., 2022	Multimodal profiling of lung granulomas in macaques reveals cellular correlates of tuberculosis control	*Immunity*	Macaques	It define the cellular ecosystems within TB lung granulomas in which Mtb is controlled.	(75)
Akter, Sadia et al., 2022	*Mycobacterium tuberculosis* infection drives a type I IFN signature in lung lymphocytes	*Cell reports*	Mouse	It identify transcriptional signatures in Mtb-infected murine lungs using single-cell RNA sequencing.	(76)
Xu, Yuzhong et al., 2022	Comprehensive identification of immuno-related transcriptional signature for active pulmonary tuberculosis by integrated analysis of array and single cell RNA-seq	*The Journal of infection*	Human	It propose that the expression of ADM in peripheral blood could be used as a novel biomarker for differentiating TB with LTBI and HC.	(77)
Chen, Qianqian et al., 2022	Characteristics of alveolar macrophages in bronchioalveolar lavage fluids from active tuberculosis patients identified by single-cell RNA sequencing	*Journal of biomedical research*	Human	It demonstrated the characteristics of alveolar macrophages from BALF in active TB patients by using scRNA-seq.	(78)
Shao, Ming-Ming et al., 2022	T Cell Receptor Repertoire Analysis Reveals Signatures of T Cell Responses to Human *Mycobacterium tuberculosis*	*Frontiers in microbiology*	Human	It describes the cloning and transcription profile of αβT cell subsets in tuberculosis patients.	(79)
Oelen, Roy et al., 2022	Single-cell RNA-sequencing of peripheral blood mononuclear cells reveals widespread, context-specific gene expression regulation upon pathogenic exposure	*Nature communications*	Human	It aims to disentangle the gene expression and gene regulatory processes that are driven by differences in genetics and/or pathogen exposures, and that could explain how interindividual differences can contribute to disease risk.	(80)
Wang, Yi et al., 2023	Systemic immune dysregulation in severe tuberculosis patients revealed by a single-cell transcriptome atlas	*The Journal of infection*	Human	It depicts a high-resolution transcriptomic landscape of peripheral immune cells during disease progression of active TB, and observed critical changes to severe TB patients.	(81)
Musvosvi, Munyaradzi et al., 2023	T cell receptor repertoires associated with control and disease progression following *Mycobacterium tuberculosis* infection	*Nature Medicine*	Human	T cell receptors related to the control and disease progression of Mycobacterium tuberculosis infection were extensively screened by using scTCR-seq.	(82)
Yang, Xinting et al., 2023	Single-cell profiling reveals distinct immune response landscapes in tuberculous pleural effusion and non-TPE	*Frontiers in immunology*	Human	Reveal the landscape of immune cells in pleural effusion, and reveal the unique local immune response in TPE and non-TPE.	(83)

### 3.1 The immune state and characteristics of lung

Macrophages are the most important host cells infected by *Mtb*. Two main macrophage groups have been identified in the lung : tissue-resident alveolar macrophages (AMs) and monocyte-derived interstitial macrophages (IMs). In the airways, *Mtb* first encounters alveolar macrophages (AMs), which present a permissive niche for infection establishment (84). After the infected AM migrates to the interstitial lung, *Mtb* will infect more other types of macrophages. During the period of tuberculosis infection, different phenotypes or polarization states of macrophages may affect the growth of bacteria. There are three subtypes of pulmonary macrophages identified from tuberculosis patients, and the expression levels of genes related to growth, metabolism, and hypoxia in these macrophages are different (67). Therefore, it is important to understand the functional heterogeneity of the host macrophage population in controlling or promoting bacterial growth.

The functional identification map of pulmonary macrophage subsets in mice infected with *Mtb* was constructed by scRNA-seq technology. It found the functional heterogeneity between alveolar macrophages and interstitial macrophages after *Mtb* infection. It also shows that the main macrophage population in the lung is epigenetically limited in its response to infection. ATAC-seq results showed that the infection of *Mtb* will result in the induction of similar functional phenotypes in the host macrophage phagocytes (71). Another study revealed the characteristics of alveolar macrophages in bronchoalveolar lavage fluid (BALF) of patients with active tuberculosis. It focuses on the macrophage subgroups related to inflammatory function, and nine macrophage subgroups have been identified, four of which are in the M2 polarization state. At the same time, it was found that the interaction between macrophages and other cells was enhanced after infection (78). In a word, it is feasible to use scRNA-seq to determine the way of bacterial growth control in the macrophage subgroup.

Meanwhile, it is important to explore the immune cell reaction at the infected site, especially the cell proportion, the activation state of cell subsets, and the interaction of immune cells. The lung immunity of rhesus monkeys with latent tuberculosis and active tuberculosis was studied. It was found that NK cells expressing CD27^+^ in the lung aggregated during the latent period of tuberculosis. During active pulmonary tuberculosis infection, plasma-like dendritic cells (pDCs) and activated T cells will flow into the lungs (74). In the mouse model, the infection of *Mtb* drives the characteristics of type I IFN in lung lymphocytes and causes the heat shock response of NK cells. Lymphocyte antigen 6 complex A site (Ly6A) was identified as a marker of lymphocyte activation, and it was found that Ly6A was up-regulated in activated lymphocytes after infection (76).

### 3.2 The granuloma architecture and molecular spatial distribution

The pathological sign of *Mtb* infection is the formation of granuloma in the lungs. It is a key site for the interaction between host and pathogen, which can limit or promote the survival of bacteria and is composed of a mixture of immune and non-immune cells (85–87). *In-situ* sequencing was used to analyze pulmonary granulomas in mice at different time points after *Mtb* infection. It was found that different regions of granulomas could be defined according to the frequency and distribution of transcripts. The different immune landscape of granuloma depends on the time after infection, pathological characteristics, and the proximity to bacteria. The necrosis center may contain transcripts related to immunosuppression (88). Exploring the formation and maintenance mechanism of granuloma can help to eliminate the harmful effects of granuloma on the host and deeply understand the pathogenesis and therapeutic targets of tuberculosis.

To understand the correlation between granuloma and bacterial control, cynomolgus macaques’ granulomas were divided into 13 cell subsets. It is found that bacteria persist in granulomas rich in mast cells, endothelial cells, fibroblasts, and plasma cells. Granuloma, which drives and controls bacteria, is characterized by abundant T cells such as type 1 and type 17, which participate in the pro-inflammatory signal network involving different cell groups. Granuloma in the late stage of infection shows the functional characteristics of restrictive granuloma, which can better control the infection of *Mtb* (75). On the other hand, macrophages are the main cell type carrying *Mtb*, which exists in pulmonary tuberculosis granuloma (89). Macrophages and epithelial transformation are necessary for the formation of organized granuloma (90). Single-cell sequencing of the zebrafish-marine mycobacteria model also confirmed the basis of macrophage transformation and the diversity of cells in the granuloma. In granuloma, not only type 1 inflammatory reaction occurs but also type 1 and type 2 reactions are induced. The marker of type 2 reaction is located in the epithelial area of granuloma. Stat6 signaling mediates type 2 immune signaling, which is necessary for the formation of necrotizing granuloma and epithelial macrophages (73). It is the unknown of granuloma that is the basic reason for many difficulties in clinical treatment. However, with the change in treatment methods, most of the current research is carried out in animal models, and the contribution of related molecules and pathways in human tuberculosis remains to be explored.

### 3.3 Immune microenvironment of extrapulmonary tuberculosis

Tuberculous pleural effusion (TPE) is the second common form of extrapulmonary tuberculosis caused by *Mtb* infection, which is characterized by the influx of immune cells into the thoracic cavity (91). Its manifestations range from spontaneous absorption of benign pleural effusion to complicated pleural effusion with thickening, empyema, and even fibrosis, which may lead to continuous impairment of lung function (92). Early diagnosis is important to prevent serious complications and treatment. However, the understanding of inflammatory mediators and immune cells in local tissues is relatively backward. Therefore, it is urgent to establish early diagnosis methods and reveal local immune mechanisms. Due to the low sensitivity of other specimens and the invasive and technical difficulty of thoracoscopy, pleural effusion has become the first choice for diagnosis and mechanism research (93).

There are two studies combining single-cell TCR sequencing with scRNA-seq to explore the types, characteristics, and distribution of T cells in tuberculous pleural effusion. One of them mainly discussed the complementarity determination region 3 (CDR 3) length, hydrophobicity, and clonal expansion of T cells. It identified a new subset of polyfunctional CD4 T cells with CD1-restricted, T^+^_H_1, and cytotoxic characteristics. This research provides some new thoughts on the protective immunity against *Mtb* (79). Another research showed that the proportion and differentiation trajectory of T cell clusters in peripheral blood and pleural effusion were different. T cells in pleural effusion have high heterogeneity and unique highly amplified T cell clones. CD8^+^T cells expressing granzyme K are preferentially enriched in pleural effusion, which may be involved in the occurrence of diseases. These findings reveal the T-cell immune status of patients with tuberculous pleurisy (94). In addition to T cells, tuberculous pleurisy also involves a variety of immune cell types. The study revealed the single-cell landscape of immune cells in tuberculous pleurisy pleural effusion, and found that the immune exhaustion of NK cells and the increase of pro-inflammatory factors driven by macrophages were its important characteristics (83). In addition, it also reveals the unique local immune response in leaking pleural effusion and malignant pleural effusion. These findings will improve the understanding of the infection and immune mechanism of extrapulmonary tuberculosis.

### 3.4 The different stages of infection

Most patients infected by *Mtb* are still asymptomatic, which is called latent tuberculosis infection, and some of them will develop into active tuberculosis (95). Major challenges of tuberculosis control are the lack of biomarkers to distinguish the status of different infections, to assess the severity of diseases, and to predict disease outcomes. To make the diagnosis, more and more methods have been established besides traditional cultural techniques. The infection of tuberculosis was diagnosed by immunological methods: Mantoux tubulin skin test (TST) and interferon gamma release assay (IGRA) (96). It was found by flow cytometry that the specific CD4^+^T cells of dominant TNF-α^+^ *Mtb* were different in latent infection and active infection (97). However, even with the development of many molecular detection technologies, there are still problems such as false positive diagnosis among vaccinated people. It is necessary to know the immune factors related to disease progression to promote the immune control of *Mtb*. The higher resolution and numerous functional parameters brought by single-cell sequencing technology help us to further explore the changes of immune cell subsets and related dynamic regulation ways under different infection conditions.

It has been found that infection can change the frequency of immune cell subsets in TB by comparing the single cell data of healthy people (HC), latent tuberculosis patients (LTBI), and active tuberculosis patients (TB). Specifically, the natural killer cell subsets (CD3^-^CD7^+^GZMB^+^) are gradually depleted from HC to LTBI and TB, and it is proved that the change in the frequency of this subset can distinguish tuberculosis patients from latent patients and healthy people (69). In addition, different stages of the disease can be predicted by developing new algorithms to process the data. Using a dynamic deconvolution algorithm, it was found that compared with LTBI and the control group, NKT cells in TB patients were less activated and monocytes were more activated. LTBI individuals with a higher risk of developing active tuberculosis can be identified by different characteristics of infected monocytes (68). The above research also proves that scRNA-seq can establish significant differences between different stages of the disease according to the discovery of new cell subsets and identification of the characteristics of cell subsets.

### 3.5 Host-pathogen interaction and immune response

The innate immunity plays a role in resisting the initial infection of *Mtb*. Host coordinates various signal transduction and initiates various cellular functions, such as phagocytosis, autophagy, apoptosis, and activation of inflammatory bodies, to control or eliminate *Mtb (* 98, 99). Adaptive immunity is the key to controlling infection. The adaptive immune response to *Mtb* can be detected in 3-8 weeks after infection, which may be beneficial to the colonization of *Mtb* (100). Host-pathogen interaction will lead to different results in subsequent treatment strategies, such as cell exhaustion and immune escape, which will not be conducive to the prognosis of tuberculosis. Host-*Mtb* interaction and regulation of immune response are indispensable research in tuberculosis research.

T cell exhaustion is one of the important strategies for Mycobacterium tuberculosis to hinder adaptive immune response during infection. Exploring cell exhaustion can better design prognostic and diagnostic markers and formulate intervention measures (101). Studies have found that patients with severe tuberculosis will experience T cell exhaustion. Yi Wang et al. have successfully constructed a comprehensive immune map of the peripheral blood of active pulmonary tuberculosis patients with different severity by single-cell sequencing (81). It was found that the proportion of inflammatory immune cells (such as monocytes) increased significantly, while the abundance of various lymphocytes (such as natural killer cells and γδT cells) decreased significantly. The apoptosis induced by perforin/granzyme, FAS and XAF1 may be the reasons for the decrease in lymphocyte abundance. In addition, the immune status of severe patients is characterized by extensive immune failure of Th1, CD8^+^T, and NK cells and high cytotoxicity of CD8^+^T and NK cells.

Understanding the immune escape mechanism of *Mtb* is of great significance for the prevention, diagnosis and treatment of tuberculosis. A large number of studies have expounded the molecular mechanism (102). For example, the protein kinase G (PknG) in *Mtb* can inhibit the innate immunity of the host by binding to the host E2 protein UbcH7 through the ubiquitin-like (Ubl) domain (103). Cytoplasmic sensors cGAS, IFI204, and AIM2 recognize *Mtb* DNA, while RIG-1, MDA5, and PKR detect RNA. After that, activated cytosolic DNA/RNA sensors further induce the activation of inflammasomes or NF-κB- and IRF3-mediated innate immune pathways to regulate host anti-*Mtb* responsese (104). However, single cell sequencing technology has not been fully used in the study of immune evasion of tuberculosis. We hope that high-throughput sequencing can be combined with existing research methods in future research, so as to further clarify the immune escape mechanism.

### 3.6 Targets of drugs and vaccines

Due to the improper use of antibiotics, the evolution of *Mtb*, and co-infection with HIV, the emergence of extensively drug-resistant tuberculosis and completely drug-resistant tuberculosis is increasing (105, 106). At the same time, the drug treatment of tuberculosis has not changed. At present, it mainly includes four drugs: isoniazid, rifampicin, pyrazinamide, and ethambutol. Studies have shown that there are at least 14 candidate drugs for susceptible, drug-resistant, and latent tuberculosis in the world at the stage of clinical drug development (107). However, it is still far from enough to develop new tuberculosis drugs and prevention strategies. In recent years, only two anti-tuberculosis drugs have been developed (107, 108).

Rifampicin (RIF) was first used in the clinical treatment of tuberculosis in 1968, and it is still a first-line anti-tuberculosis drug (109, 110). Rifampicin is a semi-synthetic rifamycin drug, which can act on the rpoB gene encoding the β subunit of RNA polymerase and interfere with the synthesis of deoxyribonucleic acid and protein (111, 112). Therefore, more than 95% of RIF drug-resistant mutations are related to rpoB gene mutations (112, 113). The pharmacokinetics of RIF and rifapentine were studied by spatial metabonomics technology. The research on spatial resolution clearly and intuitively showed the metabolic pattern of drugs in animal models. The results showed that there were differences between RIF and rifapentine in penetrating into the cavity, which explained the reasons why the two drugs were not effective in treating patients with large lung cavities (114).

At present, the only licensed vaccine to prevent tuberculosis in the world is Bacille Calmette-Guérin (BCG). It has high efficacy in newborns, but with the continuous advancement of research, clinical experiments have shown that the effect of BCG in preventing tuberculosis in adolescents and adults is not satisfactory. In addition, the enhancer of BCG has not been proved to be effective (115). Through single-cell sequencing, it was found that BCG can reduce systemic inflammation and highlight the genes with non-specific protective effect of BCG. It showed the changes related to BCG-induced immune and protective effects of circulating monocytes (115, 116). T cell receptors related to the control and disease progression of *Mtb* infection were extensively screened by using scTCR-seq. These T cell antigens can be regarded as high priority targets for vaccine development (82). With the continuous development of single-cell sequencing, more therapeutic targets are gradually screened out. This provides a theoretical basis for establishing an evaluation system of tuberculosis immune protection and guiding the research and development of vaccines and drugs.

## 4 Discussion

Single-cell sequencing is changing traditional molecular biology research by establishing single-cell maps between different cells and tissues. At the same time, the mature commercial sequencing platform and a variety of developed new technologies have greatly reduced the sequencing cost, but each technology still has limitations. When some pathogens are infected, there is no 3’ poly-A tail in its mRNA, which will lead to the inability of scRNA-seq to recognize it (117). Because of the high proportion of binuclear cells in the blood of pulmonary tuberculosis patients, there are two mixed transcription groups, which will have a certain impact on data processing and analysis (118). Moreover, scRNA-seq can identify the cell subsets in the tissue, but it can’t capture their spatial distribution. In a word, there are still many challenges and opportunities in single-cell sequencing technology.

With the development of single-cell research, scientists are no longer satisfied with obtaining single information. By adding time and space information and combining with higher resolution detection technology, further development is carried out. The scRNA-seq approach combined it with other powerful approaches (multiplex of genomic, and proteomic assays, to list a few) will certainly enhance the quest to reveal some of the enigmas related to *Mtb* infection and disease in humans. Spatial transcriptomics has also played a promoting role in the related research of *Mtb* infection. Andrew J Sawyer et al. used single-cell multiple fluorescence phenotype analysis and spatial single-cell analysis algorithm to analyze tuberculosis tissue slice samples. The whole landscape map was constructed, and the classification and pathological classification of tuberculosis granuloma were systematically established. In addition to the recognized necrotizing lesions, it is also identified that there are at least four types of obvious non-necrotizing lesions in composition and space. These lesions are formed by adjacent necrotizing granulomas and tissues in the focus of immune cells (119). However, although the development of related spatial transcriptome sequencing technology has provided researchers with higher dimensional choices, spatial transcriptome technology cannot achieve the analysis of deep single-cell transcriptome resolution at present (31).

Rapid identification of bacterial phenotype, virulence and antibiotic sensitivity is important for infectious disease management. As a high-resolution tool, whole genome sequencing has been widely used in clinical diagnosis and risk management. However, at present, single-cell genome sequencing is rarely used in clinical research. The combination of single cell sequencing and whole-genome amplification can reveal the lifestyle of environmental bacteria (120). Combined with metagenomics, it can be used to identify the biosynthetic pathway of apratoxin (a potent cancer cell toxin with medicinal prospects) (121). The antigens and targets related to infection, immunity and vaccine of *Mtb* can be found by whole genome sequencing (122). Through DNA sequencing, it was found that compared with the original strain, there were gene mutations related to virulence in the strains exposed to the vaccine (123). However, we have not found that single-cell genome sequencing is used in the research related to *Mtb*. With the maturity of technology, we believe that this method is conducive to the clinical identification of a variety of bacterial infections, bacteria related to genetic susceptibility and new uncultured pathogens (124). It can also expand our understanding of host-*Mtb* interaction.

With the breakthrough of related technologies and the improvement of resolution, single-cell sequencing also provides support for the research of protein omics (125). By detecting the proteins interacting between *Mtb* and 11 kinds of STPKs, the STPKs- protein interaction network map was constructed, including 492 binding proteins and 1027 interacting proteins. It is found that PknG maintains the integrity of the cell wall through peptidoglycan (PG) biosynthesis (126). Through the combination of various omics techniques, people can further grasp the changes and characteristics of tuberculosis at multiple levels and tissues, reveal the relationship between molecules, and promote the identification of interrelated biological processes (127, 128). This also provides a new dimension for drug research and development and contributes to the prevention and control of tuberculosis.

The integration of omics technology needs many methods and tools to analyze and interpret the generated multidimensional data. Every omics has developed statistical methods to analyze its high-quality data. However, when data from different sequencing methods and platforms are integrated for analysis, the inconsistency of data characteristics may not be properly handled (129, 130). This is also a difficult point in multivariate analysis. Some studies have tried to use integrated genomics to characterize the resistance mechanism of *p*-aminosalicylic acid to multidrug-resistant tuberculosis and extensively drug-resistant tuberculosis (131). The study of integrated omics at single cell level is rare, but we think it has potential research value in understanding the gene regulation, metabolism, adaptability and pathogenicity of *Mtb*.

In a word, the complexity of cell subsets in body fluids increases with the development of advanced technology. Single-cell sequencing provides a new direction for precise treatment by exploring specific cell subsets, mining key molecules, and clarifying the mechanism of pathogen-host interaction. In the future, single-cell sequencing will be more widely used in the study of pathogenic microbial infections and other diseases.

## Author contributions

JP: Writing – original draft, Conceptualization, Writing – review & editing. ZC: Writing – original draft, Conceptualization, Writing – review & editing. XZ: Visualization, Writing – review & editing. QD: Investigation, Writing – review & editing. HZ: Investigation, Writing – review & editing. JS: Supervision, Writing – review & editing. GW: Supervision, Writing – review & editing.
