# Supplementary material for: Research progress of single-cell sequencing in tuberculosis
Source: Front Immunol. 2023 Oct 13;14:1276194. doi: 10.3389/fimmu.2023.1276194 (PMC10611525; doi:10.3389/fimmu.2023.1276194)
Supplement: Supplementary file 1 [file Table_1.docx]

**Supplementary Table 1 The development of scRNA-seq technology.**

| **Technology** | **Year** | **Developers** | **Ref（PMID）** |
| --- | --- | --- | --- |
| Single cell mRNA-Seq | 2009 | Fuchou Tang et al. | 19349980 |
| STRT-Seq | 2011 | Saiful Islam et al. | 21543516 |
| Smart-Seq | 2012 | Daniel Ramsköld et al. | 22820318 |
| CEL-Seq | 2012 | Tamar Hashimshony et al. | 22939981 |
| Smart-seq2 | 2013 | Simone Picell et al. | 24056875 |
| ATAC-seq | 2013 | Jason D Buenrostro et al. | 24097267 |
| Quartz-Seq | 2013 | Yohei Sasagawa et al. | 23594475 |
| MARS-seq | 2014 | Diego Adhemar Jaitin et al. | 24531970 |
| Drop-Seq | 2015 | Evan Z Macosko et al. | 26000488 |
| inDrop | 2015 | Allon M Klein et al. | 26000487 |
| CEL-Seq2 | 2016 | Tamar Hashimshony et al. | 27121950 |
| Div-Seq | 2016 | Naomi Habib et al. | 27471252 |
| DroNc-seq | 2017 | Naomi Habib et al. | 28846088 |
| MATQ‐seq | 2017 | Kuanwei Sheng et al. | 28092691 |
| Seq‐Well | 2017 | Todd M Gierahn et al. | 28192419 |
| Quartz-Seq2 | 2018 | Yohei Sasagawa et al. | 28846088 |
| MARS-seq2.0 | 2019 | Hadas Keren-Shaul et al. | 31101904 |
| DBiT-seq | 2020 | Yang Liu et al. | 33188776 |
| scifi-RNA-seq | 2021 | Paul Datlinger et al. | 34059827 |
| Live-seq | 2022 | Wanze Chen et al. | 35978187 |
| FIPRESCI | 2023 | Yun Li et al. | 37024957 |
